# Spiral Ganglion Neuron Projection Development to the Hindbrain in Mice Lacking Peripheral and/or Central Target Differentiation

**DOI:** 10.3389/fncir.2017.00025

**Published:** 2017-04-13

**Authors:** Karen L. Elliott, Jennifer Kersigo, Ning Pan, Israt Jahan, Bernd Fritzsch

**Affiliations:** Department of Biology, University of IowaIowa City, IA, USA

**Keywords:** ear, development, sensory epithelia, sensory neurons, auditory nuclei, Atoh1 mutation

## Abstract

We investigate the importance of the degree of peripheral or central target differentiation for mouse auditory afferent navigation to the organ of Corti and auditory nuclei in three different mouse models: first, a mouse in which the differentiation of hair cells, but not central auditory nuclei neurons is compromised (*Atoh1-cre; Atoh1*^*f*/*f*^); second, a mouse in which hair cell defects are combined with a delayed defect in central auditory nuclei neurons (*Pax2-cre; Atoh1*^*f*/*f*^), and third, a mouse in which both hair cells and central auditory nuclei are absent (*Atoh1*^−/−^). Our results show that neither differentiated peripheral nor the central target cells of inner ear afferents are needed (hair cells, cochlear nucleus neurons) for segregation of vestibular and cochlear afferents within the hindbrain and some degree of base to apex segregation of cochlear afferents. These data suggest that inner ear spiral ganglion neuron processes may predominantly rely on temporally and spatially distinct molecular cues in the region of the targets rather than interaction with differentiated target cells for a crude topological organization. These developmental data imply that auditory neuron navigation properties may have evolved before auditory nuclei.

## Introduction

Experimental tracing of developing afferent innervation from individual end organs to the brain show initially segregated projections of all major sensory organs (Fritzsch et al., 2005a) and spatio-temporal segregation of afferent projections of vestibular and cochlear endorgans to the brainstem (Fritzsch et al., 2015; Dabdoub and Fritzsch, 2016). Segregation of projections appears to develop before peripheral and central target cells differentiate (Zecca et al., 2015), suggesting that topological projections to the hindbrain arise through temporal progression of afferent development (Fritzsch et al., 2005a) or using existing diffusible factors such as Wnt's, Bmp's, and Shh that form dorso-ventral gradients (Litingtung and Chiang, 2000; Fritzsch et al., 2006; Lai et al., 2016). The mammalian vestibular afferents develop about 2 days before cochlear afferents and each projects without any apparent overlap directly to their future target nuclei (Fritzsch et al., 2015) around the time the first neurons exit the cell cycle (Pierce, 1967; Altman and Bayer, 1980). The cochleotopic map of the organ of Corti projection onto cochlear nuclei develops in embryos prior to hair cell and cochlear nucleus differentiation, apparently as a consequence of the delayed maturation of apical relative to basal spiral ganglion neurons (Ruben, 1967; Matei et al., 2005). Discrete topological projections to the cochlear nuclei are established at least 2 weeks before onset of hearing and 1 week before afferent activity is found in these neurons and adjacent cells (Wang et al., 2015), indicating that molecular cues, combined with the timing of arrival, can generate at least a crude cochleotopic map. It remains unclear how such molecular cues relate to developing hair cells and developing cochlear nuclei or whether these cues are intrinsic to the afferents or mediated in part by differentiating cochlear nucleus neurons and/or hair cells.

Recent years have seen dramatic progress in the understanding of the molecular and cellular basis of connection formation (Tessier-Lavigne, 2002; Feldheim et al., 2004; Rhinn et al., 2006). Such analyses have shown single cell molecular precision in the olfactory system (Mombaerts et al., 1996; Zou et al., 2004; Komiyama and Luo, 2006), partly based on gradients of molecules such as the eph receptors and ephrin ligands, as in retinal projections (Drescher et al., 1997; Honda, 2003; Feldheim et al., 2004; Rodger et al., 2005). Little is known about the development of the central inner ear projections (Begbie and Graham, 2001; Xiang et al., 2003), and the cochlear projection in particular (Rubel and Fritzsch, 2002; Siddiqui and Cramer, 2005), beyond data implying guidance by sempahorins/neuropilins (Gu et al., 2003; Lu et al., 2014; Coate et al., 2015), Neurod1 (Jahan et al., 2010), and other molecules such as GATA3 (Duncan and Fritzsch, 2013; Luo et al., 2013; Goodrich, 2016).

In the auditory system, both peripheral and central auditory afferent targets, the cochlear hair cells and the cochlear nuclei neurons, respectively, depend on a single gene for differentiation, the bHLH gene *Atoh1* (Bermingham et al., 1999; Fritzsch et al., 2005b; Wang et al., 2005; Rose et al., 2009). Innervation of *Atoh1* null (*Atoh1*^−/−^) ears in which no hair cells ever differentiate (Fritzsch et al., 2005a; Pan et al., 2011) or mostly disappear very early (Pan et al., 2012) show a surprising precision of afferent growth toward the absent hair cells, apparently guided by Schwann cells (Mao et al., 2014). While the initial report on Atoh1^−/−^ mice (Bermingham et al., 1999) described limited Atoh1-LacZ expression in “supporting cells” of the ear, later data showed that Atoh1-LacZ is seen in rapidly dying undifferentiated hair cells (Fritzsch et al., 2005a; Pan et al., 2012). Previous work showed expression of Atoh1 along the rhombic lip, spinal cord, and cerebellum (Bermingham et al., 2001) and detailed histology showed that precursors expressing Atoh1 lacZ remain near the rhombic lip along the hindbrain but never develop into differentiated neurons (Wang et al., 2005). The loss of differentiated auditory nuclei and related Atoh1-dependent nuclei of the brainstem in Atoh1 mutants suggests that Atoh1 mediates an essential step in the differentiation of these central neurons. While *Atoh1*^−/−^ mice lack both cochlear hair cells and cochlear nucleus neurons (Bermingham et al., 1999; Wang et al., 2005; Rose et al., 2009), they are not viable, which precludes analysis of postnatal stages. However, several mouse models exist that affect these peripheral and central targets differently. *Atoh1-cre; Atoh1*^*f*/*f*^ conditional knockout (CKO) mice lose Atoh1 expression in the periphery, and subsequently hair cells, resulting in loss of a peripheral target (Pan et al., 2012); whereas they retain Atoh1 centrally due to lack of recombination in the hindbrain with this specific cre line that retains neurons needed for breathing (Rose et al., 2009). In contrast, in *Pax2-cre; Atoh1*^*f*/*f*^ CKO mice, the onset of Pax2 in the auditory nuclei may result in a delayed loss of auditory nuclei neurons (Ohyama and Groves, 2004) due to delayed recombination both also due to loss of afferents and subsequent loss of cochlear nucleus neurons (Levi-Montalcini, 1949; Rubel and Fritzsch, 2002). Thus, while the peripheral target does not develop as in Atoh1 null mice (Pan et al., 2011), a central, but reduced, target for the remaining auditory afferents exists for some time in *Pax2-cre; Atoh1*^*f*/*f*^ CKO mice allowing to evaluate how reduced size of cochlear nuclei affects cochleotopic projections.

Here we ask what effect Atoh1-mediated differentiation of hair cells and cochlear nucleus neurons has on guiding inner ear afferents to their central targets. Using mutant mice, we tested whether inner ear afferents rely on peripheral and central targets for navigation consistent with developmental evidence (Fritzsch et al., 2005a). Our data suggest that afferents can home in on non-differentiated targets, indicating some degree of independence of afferent projections from both cochlear nucleus neurons or hair cell differentiation.

## Methods

### Mice and genotyping

All animal work was conducted according to the Care and Use of Laboratory Animals. All animal procedures were approved by the University of Iowa Institutional Animal Care and Use Committee (IACUC) (ACURF #1103057).

*Atoh1*^−/−^ mice were bred from heterozygotes as previously described (Bermingham et al., 1999; Fritzsch et al., 2005a). These mice carried the *LacZ* reporter gene in place of the Atoh1 coding sequence, allowing us to visualize the development and disappearance of precursors (Fritzsch et al., 2005a, 2006). To generate *Tg*(*Atoh1-cre); Atoh1*^*f*/*f*^ CKO mice (Pan et al., 2012), mice carrying the *Atoh1-cre* transgene (Matei et al., 2005) were bred with mice carrying floxed *Atoh1* (Shroyer et al., 2007; Maricich et al., 2009). The CKO mutants are viable and were obtained at expected Mendelian ratios for all stages. To generate *Tg(Pax2-cre); Atoh1*^*f*/*f*^ CKO mice (Pan et al., 2011), mice carrying the *Pax2-cre* transgene (Ohyama and Groves, 2004) were bred with mice carrying floxed Atoh1 (Shroyer et al., 2007; Maricich et al., 2009). The CKO mutants were viable and can live up to 1-month old.

Mice were genotyped using PCR analysis of DNA obtained from tails. The *Atoh1-cre* and *Pax2-cre* transgenes were detected by *cre*-specific primers (forward: 5′-CCT GTT TTG CAC GTT CAC CG-3′ and reverse: 5′-ATG CTT CTG TCC GTT TGC CG-3′), which generated a 280 bp product. Two internal control primers were included in the PCR reaction that produced a 330 bp product (forward: 5′-CTA GGC CAC AGA ATT GAA AGA TCT-3′ and reverse: 5′-GTA GGT GGA AAT TCT AGC ATC ATC C-3′). The *Atoh1* allele-specific primers (forward: 5′-AGC GAT GAT GGC ACA GAA G-3′ and reverse: 5′-GAA GTC AGG TCG TTG CTA AC-3′) generated a 300 bp product from the wild-type Atoh1 coding region and a 500 bp product from the floxed allele.

All postnatal mice and pregnant females for collecting embryos were anesthetized by injection of a lethal dose of Avertin (1.25% of 2.2.2-tribromoethanol at a dose of 0.025 ml/g of body weight) and then perfused with 4% paraformaldehyde (PFA) in 0.1 M phosphate buffer (pH 7.4) using a peristaltic pump. Heads were isolated and fixed for at least 24 h in 4% PFA before further processing.

For all conditions, both ears of at least three mutants and littermate controls from at least two litters were examined for the stages indicated in results for each phenotype. Between mutant variation was so limited relative to control animals allowing the generation of compelling qualitative evidence displayed in our data.

### LacZ

Beta-galactosidase detection to express LacZ was run as previously described (Matei et al., 2006). The reaction product remains in undifferentiated cells that upregulate Atoh1 prior to their degeneration (Fritzsch et al., 2005a).

### Lipophilic dye labeling

Heads of mice were cut sagittally at the midline. For *Atoh1-cre; Atoh1*^*f*/*f*^ and *Pax2-cre; Atoh1*^*f*/*f*^ mice, lipophilic dye-soaked filter strips were implanted into the cochlea modiolus and the vestibular utricle, anterior and horizontal canal crista. The cochlea dye applications aimed for the center of the coiled cochlea to label most if not all spiral ganglia while avoiding labeling the adjacent saccule. Vestibular afferent labeling was targeted toward the largest number of afferents while keeping the saccule and posterior canal fibers unlabeled. For *Atoh1*^−/−^ mice, small pieces of lipophilic dye-soaked filter strips (Fritzsch et al., 2005c) were implanted at the apex and base of the cochlea using landmarks such as the round window for the base and the tip of the coiled cochlea for the apex. All injections were verified using whole mounted ear preparations as previously described (Maklad and Fritzsch, 2003) and only preparations with confirmed applications as intended were further analyzed.

### Imaging

Brains and ears were micro dissected, mounted on a slide with 100% glycerol as previously described (Fritzsch et al., 2016), and viewed using a Leica SP5 confocal microscope. Stacks of images were processed using Leica LIF software, combined into plates using Corel Draw. Aoth1-LacZ reactions were flat mounted in glycerol and imaged using a Nikon upright microscope with Metamorph software for image processing.

## Results

### *Atoh1* is necessary for neuronal precursor expansion but not specification

**Figure 1:**
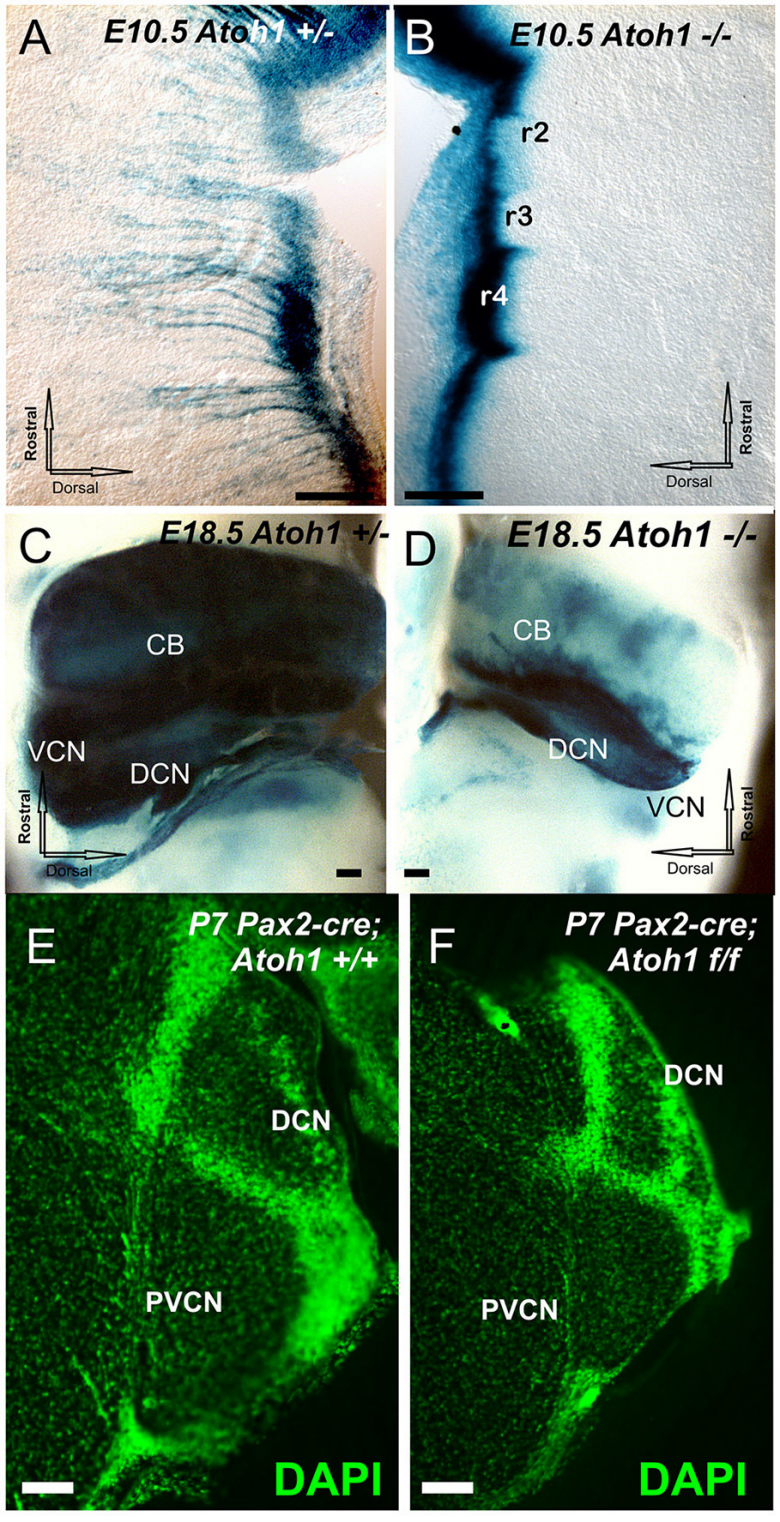
**The distribution of Atoh1 LacZ is shown by ß-galactosidase staining in *Atoh1* heterozygous (*Atoh1*^+/−^; A,C)** and *Atoh1*^−/−^ littermates (*Atoh1*^−/−^; **B,D**) at embryonic day **(E)** 10.5 **(A,B)**, E18.5. **(C,D)** and effects of Pax2-cre mediated deletion of Atoh1 **(E,F)**. Between E10.5 and E18.5 the rhombic-lip shows migratory cells to the isthmus, pons, cerebellum, and cochlear nuclei in *Atoh1*^+/−^ **(A,C)** but not in *Atoh1*^−/−^**(B,D)**. Consistent with previous detailed analysis (Wang et al., 2005; Rose et al., 2009) there is absence of auditory nuclei in the *Atoh1* null mice **(C,D)** leaving only the Atoh1-LacZ stain along the rhombic lip. Comparison of sections at cochlear nerve entry of control and Pax2-cre, Atoh1 f/f. shows profound reduction likely due to an unclear mix of afferent fiber loss and direct and indirect degeneration of cochlear nucleus neurons **(E,F)**. CB, cerebellum; DCN, Dorsal cochlear nuclei; DCN, dorsal cochlear nucleus; PVCN, postero-ventral cochlear nucleus; VCN, Ventral cochlear nuclei; VIII, VIII nerve root. Bar indicates 100 μm.

We first assessed the fate of central Atoh1 expressing neurons, prior to the already existing data starting at E12.5 (Wang et al., 2005; Rose et al., 2009) to correlate Atoh1 null effects with the earliest reported cochlear nucleus neurons exiting the cell cycle at E10 (Pierce, 1967), 1 day ahead of spiral ganglia and 2 days ahead of hair cells (Altman and Bayer, 1980; Matei et al., 2005). To assess the role of Atoh1 in precursor formation and/or expansion, we used *Atoh1-LacZ* heterozygous and null mice to investigate regions where Atoh1 is expressed. Absence of *Atoh1* does not affect the initial formation of the precursors along the rhombic lip (Figures 1A,B) but Atoh1 is required for precursor expansion in the cerebellum (Pan et al., 2009) as well as in the various migratory streams, which form incompletely and transiently in the null mice (Figures 1A,B). These data expand previous work to an earlier stage (E10.5) and confirm previous work on full or rhombomere-specific deletion of *Atoh1* in later stages (Wang et al., 2005; Maricich et al., 2009; Rose et al., 2009) showing near complete loss of any differentiated cell in the cochlear nuclei (Figures 1C,D). Beyond undifferentiated precursors bound to degenerate via apoptosis no central or peripheral targets for spiral ganglia develop in *Atoh1* null mice.

### *Atoh1-cre; Atoh1*^*f*/*f*^ CKO mice, lacking a peripheral target, reveal normal central projections

**Figure 2:**
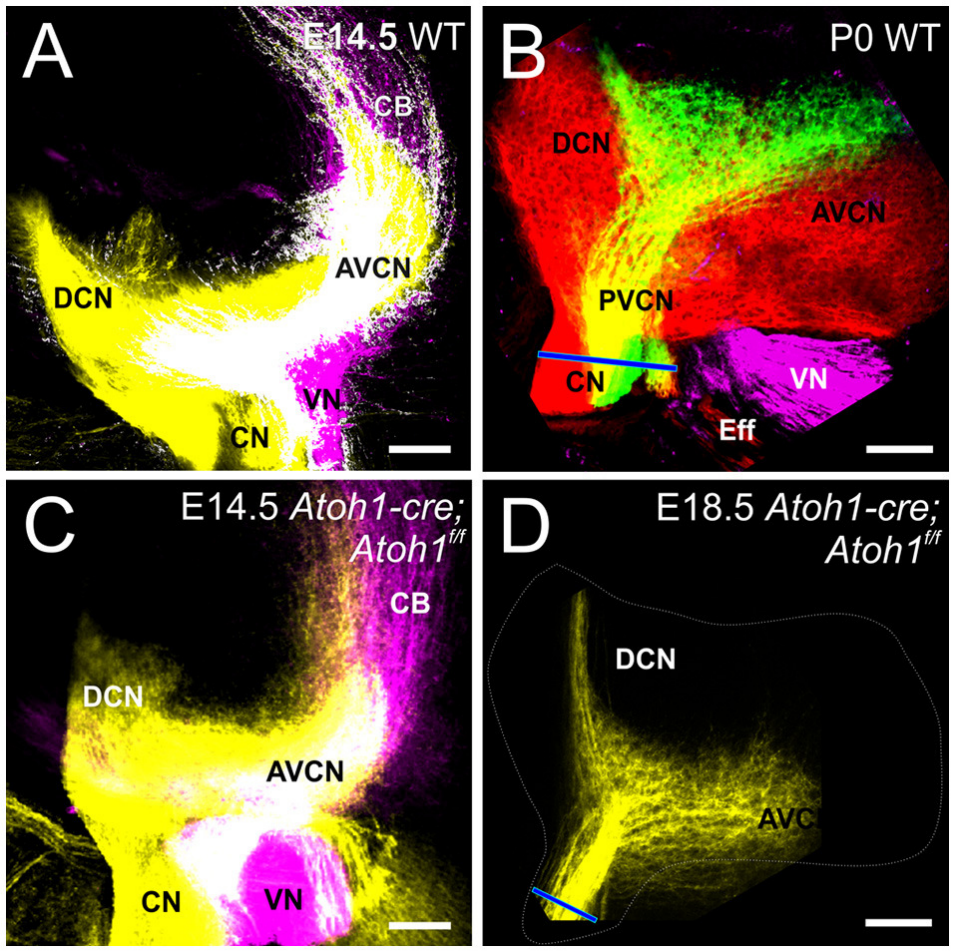
**Cochlear and Vestibular projections segregate centrally in the absence of differentiation of hair cells**. At E14.5, dye injection into the cochlea and vestibular endorgans show segregation in the cochlear nucleus and vestibular nucleus in control **(A)** and in *Atoh1-cre Atoh1*^*f*/*f*^ mice **(C)** that lack differentiated hair cells. Cochlear projections remain in cochlear nuclei at E18.5 **(D)**, but are smaller compared to control **(B)**. Blue line in **(B,D)** indicates cochlear nerve diameter as an indicator of reduction spiral ganglion afferents. Cochlear afferents are colored yellow (or separately as red and green when apex and base are individually labeled, respectively), vestibular afferents are colored magenta. AVCN, Anteroventral Cochlear Nucleus; PVCN, Posteroventral Cochlear Nucleus; DCN, Dorsal Cochlear Nucleus; CN, Cochlear Nerve; VN, Vestibular Nerve; CB, Cerebellar fibers; Eff, Efferents; IX, Glossopharyngeal. Bars indicate 100 μm.

*Atoh1-cre; Atoh1*^*f*/*f*^ “self-terminating” mice use an Atoh1 enhancer element driven Cre expression (Matei et al., 2005) to recombine the floxed Atoh1 alleles after an initial normal expression of Atoh1. Many hair cells initially differentiate but die over time due to lack of Atoh1 (Pan et al., 2012). Cochlear nucleus neurons are known to critically depend on Atoh1 for viability and can be eliminated in a rhombomere-specific loss using proper cre drivers (Maricich et al., 2009). Also, Atoh1 “self-terminating” mutants can survive into adulthood (P36), though it remains unclear how many cochlear nucleus neurons die and at which time point, as some neurons involved in breathing differentiate that are dependent on Atoh1 in these conditional Atoh1 mutants (Rose et al., 2009). With the uncertainty about the degree of viability of cochlear nucleus neurons in mind, we asked in these mutants whether auditory afferents require information from the periphery to segregate from vestibular afferents centrally. Using lipophilic dye injections, we show that auditory afferents segregate from vestibular afferents in the hindbrain as early as E14.5 (Figure 2C), as in controls (Figure 2A) indicating that the periphery is not necessary for central pathfinding as timing of segregation seems not to differ from normal timing of segregation (Fritzsch et al., 2015). Spiral ganglion projections remain confined to the cochlear nuclei but are much less dense compared to control animals due to the massive loss of many spiral ganglion neurons as hair cells die (Pan et al., 2012) and the organ of Corti dedifferentiates (Figure 2D vs. Figure 2B).

### *Pax2-cre; Atoh1*^*f*/*f*^ CKO mice, lacking a peripheral target and having a delayed loss of a central target, albeit reveal normal central projections

**Figure 3:**
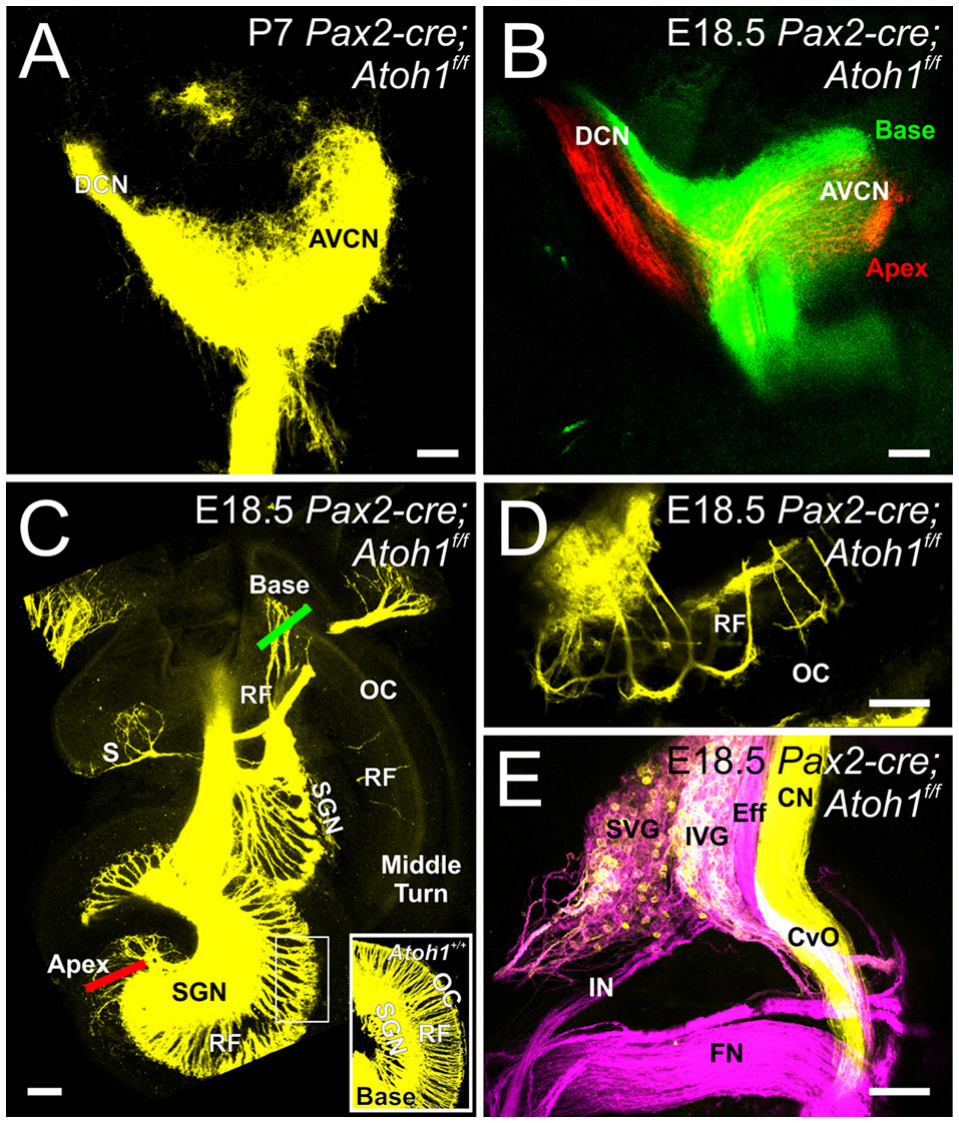
**Inner ear projections segregate when hair cells at the periphery do not differentiate and when there is a possible delayed loss of cochlear nucleus neurons**. Dye injected into the cochlea reveal that cochlear afferents remain confined to the cochlear nucleus at P7 **(A)**. Dye injected into the base (green) and apex (red) of the cochlea reveals segregation of these afferents within the cochlear nucleus in E18.5 *Pax2-cre Atoh1*^*f*/*f*^ mice **(B)**. Dye injected centrally into the hindbrain reveals afferent projections to vestibular endorgans and the cochlea **(C)**. Red and Green bars indicate placement of dye for apical and basal injections, respectively, shown in **(B)**. Note that no afferents extend to the organ of Corti in the middle turn with few afferents reaching the basal organ of Corti and expanding over the apex organ of Corti **(C)** as compared with controls that have afferents to all regions of the organ of Corti, including the base (inset). All epithelia are innervated despite lack of hair cell differentiation, with the most profound loss of afferents being in the saccule (S). Apical dye injection into the cochlea shows afferent and efferent labeling next to but not into the organ of Corti **(D)** at the approximate position indicated by the box in C. Central application of dye into vestibular nucleus/efferents in rhombomere 4 and to the cochlear nucleus/vestibular nucleus in rhombomere 5 show labeling in the facial nerve (FN), the vestibular and cochlear efferents (Eff) including efferent fibers in the commissure of van Oort (Cvo), the distinctly labeled cochlear nerve (CN, yellow) and a mix of vestibular neurons labeled by either dye application in superior and inferior vestibular ganglion (SVG, IVG) **(E)**. Cochlear afferents are colored yellow (or separately as red and green when apex and base are individually labeled, respectively), vestibular afferents are colored magenta. AVCN, Anteroventral Cochlear Nucleus; DCN, Dorsal Cochlear Nucleus; SGN, Spiral Ganglion Neurons; RF, Radial Fibers; SVG, Superior Vestibular Ganglion; IVG, Inferior Vestibular Ganglion; IN, Intermediate nerve. Bars indicate 100 μm.

In contrast to Atoh1-cre; *Atoh1*^*f*/*f*^ “self-terminating” mice that initially form partially differentiating hair cells that die as the animals mature, *Pax2-cre; Atoh1*^*f*/*f*^ CKO mice never have differentiated hair cells (Pan et al., 2011) but survive for at least 30 days. In addition to the absence of any differentiated hair cells in the ear, these mice may also experience a delayed loss of central target neurons due to the delayed upregulation of Pax2-cre in the auditory nuclei (Ohyama and Groves, 2004). This possibility of likely known defects caused in auditory nuclei after Cre-mediated ablation (Maricich et al., 2009) is compounded by loss of afferents (Pan et al., 2011) and afferent loss mediated degeneration of auditory nucleus neurons (Rubel and Fritzsch, 2002) resulting in a marked reduction of cochlear nucleus size (Figures 1E,F). Since this possible delayed loss may occur after the E14.5 data point we used in the *Atoh1-cre*; *Atoh1*^*f*/*f*^ “self-terminating” mice, we confirmed in this mouse model that complete vestibular and cochlear afferent segregation is retained in the hindbrain at E18.5 in *Pax2-cre; Atoh1*^*f*/*f*^ CKO mice (Data not shown). In fact, cochlear projections remain confined within the cochlear nucleus at P7 without aberrant targeting (Figure 3A). Since cochlear afferents targeted the embryonic auditory nucleus, we analyzed the central projection of cochlear afferents after dye insertion into the apex and base, respectively, to determine whether a normally developed central target was necessary for cochleotopic segregation in neonates. As previously described for control animals (Fritzsch et al., 2015), analysis of these central projections using lipophilic dyes results in a discrete, non-overlapping projection of apical and basal fibers to the cochlear nucleus complex at E18.5 (Figure 3B). Likewise, despite loss of Atoh1-mediated hair cell differentiation there is a reduction in, but not a deviation from, normal projection at the level of afferents to the ear (Figure 3C) as well as navigation of afferents and efferents from the brainstem (Figure 3E). The afferents in the apex and basal tip form interacting fiber bundles/loops adjacent to the organ of Corti area without entering into the organ of Corti (Figure 3D). However, there is no overshooting of the cochlea as described in Schwann cell mutant mice (Mao et al., 2014).

These data suggest that the initial auditory and vestibular afferent segregation and cochleotopic afferent segregation in mouse embryos is independent of hair cell development and may not depend on formation/maintenance of a normally developed central target either, consistent with previous reports on rhombomere-specific cochlear nucleus deletions (Maricich et al., 2009). In contrast to the rhombomere specific loss of cochlear nucleus neurons leading to near normal projection simply by extending to normal areas of the cochlear nucleus, we show here that despite reduction in size the cochleotopic projection is scaled accordingly.

### *Atoh1*^−/−^ mice, lacking both peripheral and central targets, reveal proper segregation, and cochleotopic projections into the hindbrain

**Figure 4:**
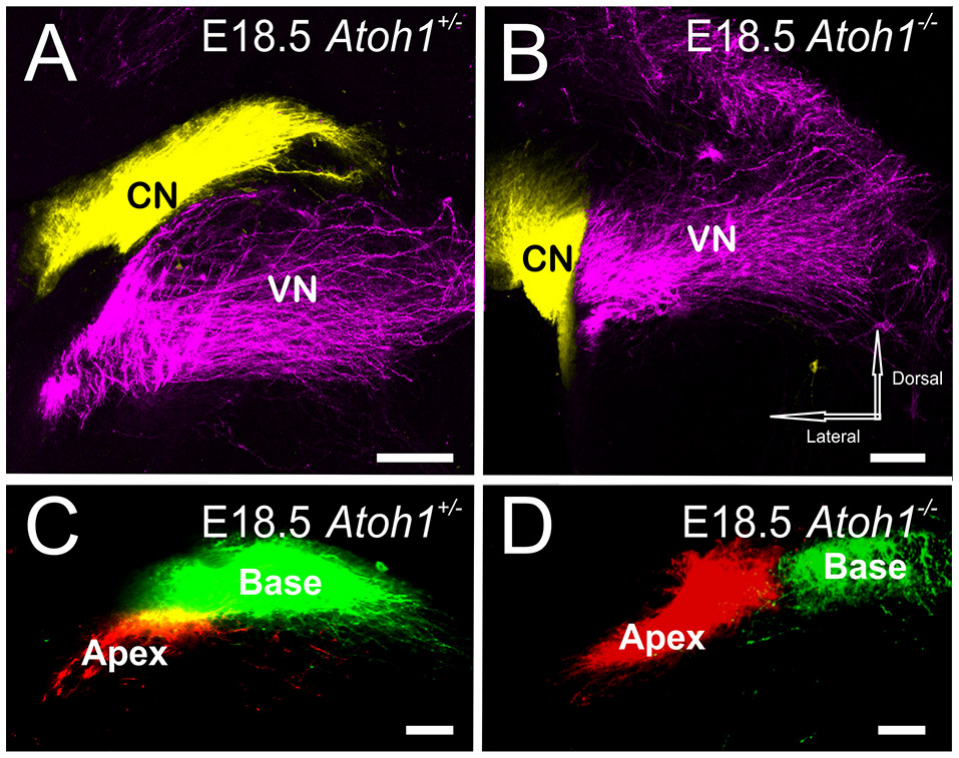
**Central projection of inner ear afferents remains segregated even if neither hair cells nor cochlear nuclei develop**. Dye injected into the cochlea and vestibular endorgans show segregation in the cochlear nucleus and vestibular nucleus in both E18.5 *Atoh1* heterozygous **(A)** and *Atoh1*^−/−^ mice **(B)**. Dye inserted into apex and base of the cochlea show fibers projecting to distinct medial and lateral divisions of the cochlear nuclei in both E18.5 *Atoh1* heterozygous **(C)** and *Atoh1*^−/−^ mice **(D)**. Cochlear afferents are colored yellow (or separately as red and green when apex and base are individually labeled, respectively), vestibular afferents are colored magenta. CN, Cochlear nucleus; VN, Vestibular nucleus. Bar indicates 100 μm.

While *Atoh1-LacZ* positive cells still form in the rhombic lip (Figure 1), there is no overt differentiation of these cell into cochlear nucleus neurons in the absence of Atoh1 (Wang et al., 2005; Maricich et al., 2009) at the time afferents are reaching the area of cochlear nuclei differentiation (E12.5; Fritzsch et al., 2015). As in the other *Atoh1* mutants, our data show segregation of vestibular afferents from cochlear afferents is complete in *Atoh1*^−/−^ and this is despite the fact that cochlear afferents have neither a peripheral nor a central differentiated target whereas the vestibular afferents only miss the peripheral target (Figures 4A,B). In addition, the overall topology of cochleotopic afferent fiber projections from the ear to the cochlear nuclei in the *Atoh1*^−/−^ mice shows a segregation of base versus apex (Figures 4C,D) as in control animals (Figure 2B) or animals that initially lose only the peripheral development (Figure 3B). In summary, our data show that despite lack of differentiated peripheral AND central target cells, cochlear afferents do not expand to nearby differentiated vestibular nuclei and develop a crude cochleotopic projection to the “cochlear nuclei” area.

## Discussion

The results presented here from various *Atoh1* mutants demonstrate that neither peripheral nor central target cell differentiation is necessary for cochlear afferent segregation from vestibular afferents or for some degree of a cochleotopic segregation. Cochleotopic connections from specific areas of the cochlea to the cochlear nuclei are the basis for tonotopic representation of the spiral ganglion projection and thus for perception of different sound frequencies (Rubel and Fritzsch, 2002; Ryugo et al., 2005). Tonotopic projections were hypothesized to develop as a consequence of activities around the afferents (Wang et al., 2015). However, afferents that are neither connected to a differentiated organ of Corti (Bermingham et al., 1999; Pan et al., 2011) nor to differentiated auditory nuclei (Wang et al., 2005; Rose et al., 2009; Figures 1, 4), have a near normal apical/basal segregation in both the anterior and posterior aspects of the cochlear nuclei (Figure 4). The degree of segregation is comparable to other conditional *Atoh1* mutations with some retention of cochlear nuclei (Figures 2, 3) and the control animals (Fritzsch et al., 2015) indicating scalability of cochleotopic projections. Since there is no gross topographic error even in *Atoh1*^−/−^ mice of afferents that have neither a central nor a peripheral cellular target, this suggests that the molecular basis of afferent targeting restricts cochlear afferents to the area of the cochlear nuclei, even if this area lacks fully differentiated target cells.

Combined, these data support the idea that cochlear afferent projections are specified by means that require either the temporal pattern of development of spiral ganglion neurons (basal turn neurons exit the cell cycle prior to apical turn; Ruben, 1967; Fritzsch et al., 2005a) or afferents growing to the hindbrain can navigate in gradients of diffusible factors that specify also areas of nuclear differentiation (Fritzsch et al., 2006; Lai et al., 2016). Clearly, neither the differentiated hair cells nor the differentiated cochlear nucleus neurons are necessary to inform cochlear afferents to form a crude cochleotopic map. Consistent with our data are data that show that central projections of the cochlea develop in a topographical fashion prior to the onset of hearing (Leake et al., 2002), even in cases where hair cells form and are later lost (Xiang et al., 2003) or Schwann cells are eliminated, resulting in disorganized peripheral projections (Mao et al., 2014). It seems likely that this topographically-restricted projection can develop based on temporal and spatial distinct expression of unknown molecules and this is basis for the late refinement of auditory connections in congenitally deaf mammals using electrical stimulation (Ryugo et al., 2005; Vollmer et al., 2005). Over time, various molecules may act as axon guidance cues such as Wnt, Shh (Stoeckli, 2006), neuregulin-1 (Lopez-Bendito et al., 2006), BMPs (Butler and Dodd, 2003), FGF receptors (McFarlane et al., 1996), robo and slit (Kim et al., 2015), and ephrins (Siddiqui and Cramer, 2005). These unknown factors can guide afferents from transplanted ears to reach vestibular nuclei (Elliott et al., 2015a) in the absence of any other pathfinding cues and are likely diffusible factors such as Wnt's (Lyuksyutova et al., 2003) or BMP's (Miguel-Aliaga et al., 2004). Future work will need to reveal how timing of differentiation (Fritzsch et al., 2005b) and patterns of expression of these guidance molecules combine to govern the development of the cochleotopic projection in mutants that lack selectively an organ of Corti and/or cochlear nucleus neurons through conditional deletion of Atoh1 in the organ of Corti or the hindbrain.

Fibers not only need to find their target, neurons need to interact with target cells to ensure their survival through the neurotrophic support molecules released from target cells (Huang and Reichardt, 2001; Fritzsch et al., 2004; Stoeckli, 2006). Absence of cochlear nuclei compromises viability of a small percentage of afferent fibers (Maricich et al., 2009), whereas absence of hair cells eliminates over 90% of all spiral ganglion neurons near term (Fritzsch et al., 2005a) except for areas with limited neurotrophin expression (Matei et al., 2006; Pan et al., 2011). Afferent fibers are, in many cases, necessary for the proper development of their target cells (Akins and Biederer, 2006; von Bartheld and Fritzsch, 2006). Auditory nuclei depend on proper afferent innervation for differentiation and survival (Levi-Montalcini, 1949; Rubel and Fritzsch, 2002) and hair cells require innervation for long-term maintenance (Kersigo and Fritzsch, 2015). This can extend to other cells if afferent input loss is early enough to capture the critical phase of dependency (Elliott et al., 2015b). Interdependence of peripheral innervation and their target cells, combined with a dependency of central target nuclei on afferent innervation, is a general feature of almost all sensory systems (von Bartheld and Fritzsch, 2006) and may in part relate to the ubiquitous afferent segregation in overlapping projections of eyes (Constantine-Paton and Law, 1978) and ears (Elliott et al., 2015a). To ensure proper support of those target neurons and hair cells, afferents need to navigate prior to target cell differentiation to reach these cells for the proper support during onset of differentiation. Our data show that this navigation is indeed independent of differentiated target cells and already properly targeted afferents can provide support to those neurons as they differentiate.

These data have implications on the evolution of the vertebrate auditory system, which has mostly focused on the terrestrial middle ear (Reichert, 1837; Maier and Ruf, 2016) and the evolution of a hearing organ in the ear (Retzius, 1884; Fritzsch, 1987) and its transformation into the mammalian organ of Corti (Fritzsch et al., 2013; Jahan et al., 2015). Molecular data begin to shed light on the development gene regulatory networks of auditory nuclei (Fritzsch, 1991; Wang et al., 2005). Auditory nuclei appear to represent rhombomere-specific transformation of a Atoh1 expression zone extending from spinal cord to the cerebellum (Bermingham et al., 2001) by unclear molecular means that may differ between different vertebrates (Grothe et al., 2004; Hernandez-Miranda et al., 2016; Iskusnykh et al., 2016; Nothwang, 2016). Our data imply auditory afferent projections could have evolved prior to auditory nuclei evolution. Once segregation of auditory afferents from vestibular fibers was established tying afferent targeting into the diffusible factors for hindbrain and spinal cord regionalization (Hernandez-Miranda et al., 2016; Lai et al., 2016), developing auditory nuclei could receive necessary support by auditory afferents, something that is of much less importance during development in the vestibular nuclei likely due to multiple inputs (Levi-Montalcini, 1949; Fritzsch, 1990).

## Author contributions

BF and KE wrote the paper; IJ, NP, and JK contributed images and edited the paper.

### Conflict of interest statement

The authors declare that the research was conducted in the absence of any commercial or financial relationships that could be construed as a potential conflict of interest.
